# Clinical Outcome of Kidney Transplant Recipients with C1q-Binding De Novo Donor Specific Antibodies: A Single-Center Experience

**DOI:** 10.3390/jcm12134475

**Published:** 2023-07-04

**Authors:** Smaragdi Marinaki, Angeliki Vittoraki, Stathis Tsiakas, Ioannis Kofotolios, Maria Darema, Sofia Ioannou, Kalliopi Vallianou, John Boletis

**Affiliations:** 1 Clinic of Nephrology and Renal Transplantation, National and Kapodistrian University of Athens Medical School, Laiko Hospital, 11527 Athens, Greece; 2 Immunology Department, National Tissue Typing Center, General Hospital of Athens “G. Gennimatas”, 11527 Athens, Greece

**Keywords:** kidney transplant, donor specific antibodies, complement, C1q binding, rejection, graft loss

## Abstract

Complement activation by HLA antibodies is a key component of immune-mediated graft injury. We examined the clinical outcomes of kidney transplant recipients with complement-fixing de novo donor-specific antibodies (dnDSA) who were followed in our center. The C1q-binding ability was retrospectively assessed in 69 patients with dnDSA and mean fluorescence intensity (MFI) values > 2000 out of the 1325 kidney transplant recipients who were screened for DSA between 2015 and 2019. Luminex IgG single antigen beads (SAB)and C1q-SAB assays (One Lambda) were used. C1q-binding dnDSA was identified in 32/69 (46.4%) of the patients. Significantly higher MFI values were observed in C1q-positive DSA (18,978 versus 5840, *p* < 0.001). Renal graft biopsies were performed in 43 of the kidney transplant recipients (62.3%) with allograft dysfunction. Antibody-mediated rejection (ABMR) was detected in 29/43 (67.4%) of the patients. The incidence of ABMR was similar among patients with C1q-binding and non-C1q-binding DSA (51.7% vs. 48.3%, *p* = 0.523). Graft loss occurred in 30/69 (43.5%) of the patients at a median time of 82.5 months (IQR 45–135) from DSA detection. C1q-binding DSA was present in more patients who experienced graft loss (53.1% vs. 35.1%, *p* = 0.152). Higher MFI values and inferior clinical outcomes occurred in most of the kidney transplant recipients with C1q-binding dnDSA.

## 1. Introduction

Progress in antibody detection techniques and immunosuppressive therapy has led to a continuous decline in early antibody-mediated rejections (ABMR) and an improvement in short-term graft survival rates in recent decades [1,2]. Despite significant advances in transplantation immunology, chronic immune-mediated injury is still recognized as the primary cause of late renal allograft loss [3,4].

The interaction between circulating alloantibodies and human leucocyte antigens (HLA) on the renal graft endothelium is a major pathogenetic mechanism of alloimmune injury [5,6,7,8,9,10]. Development of de novo donor-specific antibodies (dnDSA) has been associated with late-onset acute or chronic ABMR, allograft dysfunction, and reduced kidney graft survival [11,12]. For many years, complement-dependent cytotoxicity (CDC) assays and flow cytometry crossmatch were the standard methods for the detection of HLA antibodies [13]. The recent introduction of more sensitive solid phase immunoassays, specifically single antigen bead (SAB) assays, allows for an accurate determination of all IgG antibodies that are specific to the individual donor’s HLA antigens [14,15]. However, several studies have shown that not all kidney transplant recipients with dnDSA detected using SAB assays develop ABMR. In fact, many patients may retain stable graft function for years, with no impact on long-term graft survival [16,17,18,19,20,21,22,23]. Although SAB assays are very sensitive in identifying low levels of DSA, they cannot distinguish which phenotypic characteristics or properties of HLA antibodies are clinically relevant [24]. 

Complement fixation is essential for the pathogenesis of antibody-mediated rejection. The interaction between C1q and anti-HLA antibodies bound to the renal graft endothelium results in the activation of the classical pathway, leading to cleavage of the complement molecules C3 and C5. The breakdown products, C3a and C5a, serve as potent chemotactic factors, attracting inflammatory cells to the site, which target the graft endothelium. Furthermore, C5b production results in the formation of the membrane attack complex (MAC), which causes cell lysis and tissue injury [24,25,26,27,28,29,30,31]. Determining the ability of HLA antibodies to activate complement by binding to the C1q fragment may help discern which DSAs are pathogenic to the renal graft. The newly developed C1q binding assay can potentially discriminate between complement-fixing HLA antibodies and better assess the clinical risk of rejection [32,33,34,35,36]. A limited number of studies have shown that C1q-positive DSAs may correlate with immune-mediated injury and graft loss in kidney transplant recipients [14,32,33,37,38,39].

Identifying which dnDSA may exert harmful effects on renal grafts remains a pivotal issue in the transplant community. Immunological risk stratification currently relies primarily on the determination of the mean fluorescence intensity (MFI) values of the HLA antibodies or the performance of a renal graft biopsy, which carries an associated risk of complications [40]. The aim of this study was to report the impact of de novo C1q-binding DSA in kidney transplant recipients and to assess the ability of the C1q assay to predict adverse clinical outcomes.

## 2. Materials and Methods

### 2.1. Patient and Sample Selection 

For this retrospective analysis, we initially identified 116 subjects with dnDSA out of a total of 1325 kidney transplant recipients who were screened for DSA at our histocompatibility laboratory between 2015 and 2019. The C1q binding capacity was retrospectively assessed in 69 patients with dnDSA and MFI values > 2000. All of the included patients had received a kidney graft at our renal transplant center between 1989 and 2018. Kidney transplant recipients with pre-existing antibodies before transplantation, as well as patients with dnDSA and MFI values < 2000, were excluded from the study. 

Renal graft biopsies were performed in 43 out of the 69 kidney transplant recipients upon clinical indication, specifically new-onset proteinuria and/or increase in serum creatinine levels. Rejection episodes were classified based on the current Banff classification system. A database of the 69 kidney transplant recipients, including demographic characteristics, immunological data (panel reactive antibodies, HLA typing, and SAB results), immunosuppressive therapy regimens, histological data, and renal graft function indices, was employed for the analysis. Graft loss was considered when the patient progressed to end-stage kidney disease (ESKD). 

### 2.2. Assessment of HLA Antibodies Using the Standard IgG SAB Assay 

All of the patients were routinely tested for the presence of circulating DSA using serum samples obtained at the time of transplantation and after the transplantation annually or at the time of biopsy. The presence of HLA Class I and II IgG DSA was determined using a Luminex^®^ platform and commercially available SAB kits (LAB Screen, One Lambda, Canoga Park, CA, USA) according to the manufacturers’ instructions. EDTA treatment of the sera was performed as previously published in all samples in order to prevent the prozone effect [41,42]. Normalized MFI values obtained using Fusion software were used to assign positive antibodies. For the purpose of this study, we defined positive reactions as dnDSA with MFI > 2000. The antibody reactivity against donor HLA antigens were tested for the following loci: HLA-A, -B, -Cw, -DR, and -DQ antibodies. De novo DSA was defined as the detection of DSA only in post-transplantation sera. 

### 2.3. Assessment of HLA Antibodies Using the C1q SAB Assay 

The same banked sera of the patients found to be positive for DSA using the conventional IgG-SAB assay were further tested for complement-binding ability of antibodies. Detection of the antibodies capable of fixing to the complement was performed using a C1qScreen assay (One Lambda) according to the manufacturer’s instructions. The cutoff for a positive reaction was set at a normalized MFI value of 500 or greater [12,18,43,44].

### 2.4. Statistical Analysis 

Continuous variables were expressed as means and standard deviations or medians and interquartile ranges (IQR), depending on the normality of the data distribution. Categorical values were presented as absolute values and percentages. Means and proportions were compared using the two independent samples t-test or Mann–Whitney U test and chi-square test, as appropriate. *p*-values < 0.05 were considered statistically significant. Statistical analysis was performed using IBM SPSS Statistics Version 23.

## 3. Results

### 3.1. Demographic and Laboratory Data

Sixty-nine kidney transplant recipients with dnDSA and MFI > 2000 comprised the study cohort. Their clinical and demographic characteristics are shown in Table 1. The median age (IQR) at kidney transplant was 36(20.5) years, 62.3% of the patients were male, and 65.2% of them had received a renal graft from a living donor. ABO-incompatible transplants were performed in 8.6% of the patients. Among the included 69 patients with documented dnDSA, 32 (46%) had C1q-binding dnDSA and 37 (54%) had C1q-negative dnDSA. Most patients received basiliximab and maintenance immunosuppression with mycophenolate mofetil, calcineurin inhibitor (tacrolimus or cyclosporine), and corticosteroids. Serum creatinine levels at dnDSA presentation were increased in C1q-positive patients (2.1 mg/dl versus 1.5 mg/dl, *p* = 0.005), while proteinuria did not differ significantly (0.60 g/d versus 0.36 g/d, *p* = 0.32). 

### 3.2. Characteristics of dnDSA Detected Using IgG and C1q-SAB Assays

Among the 69 kidney transplant recipients with dnDSA and MFI > 2000, 11 had Class I IgG (15.9%), 48 had HLA Class II IgG (69.5%), and 10 had both Class I and Class II IgG (14.4%) HLA dnDSA. The HLA-DQ antibodies were more frequently represented (69.5%) and were observed in most patients with HLA Class II dnDSA (89.5%). Most C1q-positive kidney transplant recipients had HLA Class II antibodies (*p*: 0.004) in our study. Moreover, subjects with C1q-positive dnDSA had significantly higher MFI values than those with C1q-negative dnDSA (median MFI 18,978 vs. 5840, *p* < 0.001). The majority of DSAs with MFIs greater than an arbitrary threshold of 6000 were found to be capable of fixing the C1q complement factor (*p* < 0.001).

### 3.3. C1q-Binding dnDSA and Clinical Outcomes

Renal graft biopsies were performed in 43 patients (62.3%) in a median time of 12 months (IQR 57) after DSA identification. The patients with C1q-binding DSA underwent a graft biopsy more often than those with C1q-negative DSA (75% vs. 51.4%, OR: 2.8, *p* = 0.046). Among the 43 kidney transplant recipients who underwent a graft biopsy, 29 (67.4%) were diagnosed with ABMR within a median time of 19 months (IQR 68) after dnDSA presentation. The incidence of ABMR was similar between the patients with C1q-binding and non-C1q-binding DSA (51.7% versus 48.3%, *p*: 0.523). Graft loss occurred in 30 (43%) out of 69 kidney transplant recipients at a median time of 82.5 months (IQR 90) from DSA detection. Biopsy-proven ABMR was the cause of renal graft loss in 15 (51.7%) of the patients. However, in eight (26,6%) of the kidney transplant recipients who lost their renal graft, no renal graft biopsy was performed. The kidney transplant recipients with C1q-binding DSA experienced graft loss more often than those with non-C1q-binding DSA (53.1% vs. 35.1%, *p* = 0.152). Higher levels of DSA (MFI > 6000) were observed in patients who were diagnosed with ABMR (*p*: 0.079) or experienced graft loss (*p*: 0.131) (Table 2). Among the kidney transplant recipients who lost their renal graft (*n* = 30), class II dnDSA was detected in 86.7% (*p*: 0.64) of them, mostly HLA-DQ (80%). Comparing the histological features, no statistically significant difference was observed between the patients with C1q-fixing and non-C1q-fixing DSA (Table 3). Chronic histologic lesions were observed more frequently in the patients with graft loss who underwent a graft biopsy. Grade 3 glomerulosclerosis (51–75%) was present in 22.7% of the patients (*p*: 0.09), moderate interstitial fibrosis/tubular atrophy (25–50%) was present in 86.4% of the patients (*p*: 0.22), and transplant glomerulopathy was present in 40.9% of the patients (*p*: 0.85). 

## 4. Discussion

Late-onset or chronic ABMR associated with the presence of dnDSA remains one of the major causes of chronic allograft injury and reduced graft survival [45,46]. Previous studies have shown that not all HLA-DSAs have the same detrimental clinical impact [47]. The introduction of solid-phase C1q binding assays has offered a new approach for stratifying transplant immunological risk by distinguishing potentially harmful complement-fixing antibodies from those that do not fix complements and exert a lower risk for mediating rejection [48]. In our study, the relationship between C1q-fixing dnDSA and transplant outcomes was examined in a cohort of 69 adults kidney transplant recipients who developed dnDSA. Patients with C1q-fixing dnDSA had higher MFI values, and the DSAs were mainly class II, in particular, HLA-DQ. Moreover, a slight increase in the incidence of renal graft loss in C1q-positive patients was observed, although this finding was not statistically significant.

Only a limited number of studies have addressed the diagnostic and prognostic value of complement-binding Luminex^®^ assays in risk stratification for ABMR or allograft dysfunction and loss [49,50]. Some researchers have suggested that the C1q-binding capacity of the DSA may only reflect the strength of the antibodies as measured by MFI values. Therefore, the C1q-SAB assays do not offer additional information for the clinical relevance of the DSA [35,51]. Recent data have also indicated that IgG1 and IgG3 subclasses are more potent complement activators, which could explain the presumed capability of C1q Luminex^®^ assays to better stratify ABMR risk compared to conventional Luminex^®^ assays [52]. Notably, C1q binding ability and IgG3 subtype were both shown to be independent risk factors for ABMR [32]. Several investigators have reported the use of complement-binding assays to predict graft outcomes. Yabu et al. [53] were the first to report the clinical significance of C1q-positive dnDSA in adult kidney transplant recipients. They showed that DSA testing with the C1q-SAB assay had higher levels of specificity for transplant glomerulopathy and graft loss than testing with the standard IgG-SAB assay. Interestingly, all the patients who developed transplant glomerulopathy tested positive for C1q-fixing antibodies. Similarly, Thammanichanond et al. [33] reported the association between C1q-positive DSA and allograft loss without statistical significance, including different histologic features. Other groups [49,54,55] have shown that complement-fixing HLA antibodies are correlated with the development of ABMR and decreased graft survival in kidney and heart transplant patients, respectively. Loupy et al. [44] have published the largest study to date, which greatly emphasizes the association between C1q-positive dnDSA and renal outcome. They compared 239 patients with non-C1q-binding dnDSA, 77 patients with C1q-binding dnDSA, and 700 patients without DSA. The five-year graft survival differed significantly between these groups: 93%, 54%, and 94%, respectively. 

Most clinical studies have investigated the role of pre-existing C1q-binding DSA in kidney transplantation [49,56,57]. Notably, different aspects of clinical utility of C1q-binding DSA were found in pre- and post-transplant settings. Although post-transplant C1q-positive DSA detection was associated with adverse clinical sequelae, as also indicated by our study, C1q-positive DSA of pre-transplant sera did not always correlate with impaired renal graft outcomes. Of note, Otten et al. reported a significant association between pre-transplant DSA and graft survival, but no clinical impact of the C1q-binding DSA [58]. Another study by Crespo et al. [59] showed that the presence of C1q-binding DSA before kidney transplants did not predict ABMR or graft loss. However, in a small cohort of hypersensitized patients who had undergone protocol kidney graft biopsies one, three, and six months post-transplantation, the C1q-binding activity of DSA was significantly associated with ABMR, while non-complement-fixing antibodies showed no predictive value [60]. Moreover, it is widely presumed that HLA antibody strength, as measured by MFI values, is associated with adverse clinical outcomes. Sutherland et al. [16] reported the clinical relevance of C1q-positive dnDSA in a pediatric kidney transplant population and found that recipients with C1q-binding DSA had significantly higher MFI values than those with C1q- negative DSA and were more likely to have peritubular capillary C4d deposition on their biopsies, acute rejection, and allograft loss. Our study also demonstrated the association between higher HLA antibody MFI values and the capacity for complement fixing. 

The presence of dnDSA post-transplantation is a well-established independent risk factor for ABMR and graft loss. The question of whether C1q-binding DSA may result in an inferior kidney allograft outcome is still unclear. According to our results, there was no association between the presence of C1q-positive dnDSA and ABMR. This could be partly explained by the small sample size of our cohort and our decision for logistical reasons to only include patients with dnDSA and MFI values > 2000 in the analysis. Another limitation of our study was that no protocol graft biopsies were performed after dnDSA detection, so it was not possible to identify the occurrence of subclinical rejection.

Complement activation is strongly implicated in the pathogenesis of ABMR. Studies regarding the clinical impact of C1q-binding HLA antibodies provide the basis for clinical trials investigating the effect of complement inhibitors, specifically eculizumab or C1 esterase inhibitor, in the management or prevention of ABMR [61]. Interestingly, Lefaucher et al. showed that the presence of circulating complement-fixing HLA antibodies was able to predict the response to rejection prophylaxis with eculizumab in recipients with pre-transplant c1q-binding DSA [62]. In light of new emerging data, the cost of the C1q-SAB assay should be weighed against the clinical value of complement-fixing DSA detection in kidney transplantation, which requires further investigation through additional clinical studies. The development and detection of potential harmful DSAs may change the clinical approach and treatment in these patients in the near future. 

## 5. Conclusions

In conclusion, the distinction between clinically relevant and non-significant dnDSA is still a major concern for transplant care. Our findings indicate that patients with C1q-positive dnDSA exhibit higher MFI values and are possibly at a greater risk of renal graft loss. In contrast with most previously published studies, no association was found between the presence of antibody-mediated rejection and the ability of newly developed DSA to fix complements. However, not all kidney transplant recipients in our cohort underwent a renal graft biopsy. The pathogenicity of dnDSA may be currently best evaluated by a renal graft biopsy until emerging non-invasive biomarkers for rejection, such as donor-derived cell-free DNA [45], become routinely available. Considering the existing literature, more prospective studies are needed to establish the C1q assay as a predictive screening tool for graft survival in kidney transplant recipients with de novo DSA.

## Figures and Tables

**Table 1:** Demographic and clinical characteristics of kidney transplant recipients with de novo C1q-positive and C1q-negative DSA.

Variable	C1q Positive	C1q Negative	*p*-Value
Patients (*n*, %)	32 (46)	37 (54)	
Donor			
Age [years, median (IQR)]	55 (14)	53 (18.5)	0.71
Living (*n*, %)	22 (69)	20 (54)	0.50
Deceased (*n*, %)	10 (31)	17 (46)	0.56
ABOi (*n*, %)	1 (3)	5 (13.5)	0.20
Recipient			
Age [years, median (IQR)]	34 (16.5)	39 (26)	0.31
Male sex (*n*, %)	23 (72)	19 (52)	0.08
First transplantation (*n*, %)	32 (100)	36 (97)	0.49
DGF (*n*, %)	6 (18)	9 (24)	0.57
DSA Class I (*n*, %)	2 (6)	9 (24)	0.03
DSA Class II %	24 (75)	24 (65)	0.86
DSA Class I + II %	6 (19)	4 (11)	0.73
Renal biopsy (*n*, %)	24 (75)	19 (51)	0.04
Creatinine at dnDSA [mg/dl, median (IQR)]	2.1 (0.8)	1.5 (0.8)	0.005
Proteinuria at dnDSA [g/24 h, [median (IQR)]	0.6 (2)	0.36 (1)	0.32
Time interval from Tx to dnDSA [months, median, (IQR)]	81 (88.2)	55 (86.7)	0.58
Induction treatment			
Basiliximab (*n*, %)	16 (50)	26 (70)	0.34
ATG (*n*, %)	6 (18.5)	4 (10.1)	0.30
Maintenance Treatment			
MPAA at dnDSA (*n*,%)	30 (93)	36 (97)	0.47
CNI at dnDSA (*n*, %)	27 (84)	30 (81)	0.76
mTOR at dnDSA (*n*, %)	4 (12)	11 (30)	0.14
GCs discontinuation at dnDSA (*n*, %)	3 (9)	13(35)	0.01

DSA: donor-specific antibodies; ABOi: ABO incompatible; DGF: delayed graft function; dnDSA: de novo donor-specific antibodies; ATG: antithymocyte globulin; CNI: calcineurin inhibitors; MPAA: mycophenolate acid analogues; mTOR: mammalian target of rapamycin, Tx: transplantation.

**Table 2:** Associations between DSA MFI values, C1q binding ability, and clinical outcomes.

DSA MFI Values	C1q Binding Ability (*n* = 32)	ABMR (*n* = 29)	Graft Loss (*n* = 30)
<6000 (*n* = 29)	3 (10.3%)	12 (41.3%)	8 (27.5%)
>6000 (*n* = 43)	29 (90.6%)	17 (58.6%)	22 (51.1%)
*p*-value	<0.001	0.079	0.131

**Table 3:** Graft loss and histologic findings of kidney transplant recipients with de novo C1q-positive and C1q-negative DSA.

	**C1q-Positive (*n* = 32)** **(*n*, %)**	**C1q-Negative (*n* = 37)** **(*n*, %)**	***p*-Value**
Graft loss (*n* = 30)	17 (53)	13 (35)	0.15
Graft biopsy (*n* = 43)	24 (75)	19 (51)	0.04
**Histologic Features**	**C1q-Positive (*n* = 24)** **(*n*, %)**	**C1q-Negative (*n* = 19)** **(*n*, %)**	***p*-Value**
ABMR	15 (62.5)	14 (74)	0.43
Transplant Glomerulopathy	8 (33)	9 (47)	0.35
IFTA (mild)	5 (21)	4 (21)	0.98
IFTA (moderate)	19 (79)	15 (79)	1.00
Glomerulosclerosis (0–25%)	11 (46)	11 (58)	0.76
Glomerulosclerosis (26–50%)	9 (37.5)	6 (32)	0.68
Glomerulosclerosis (51–75%)	4 (16.5)	2 (10)	0.52
Arteriosclerosis (mild, moderate)	17 (74)	17 (90)	0.25
Arteriosclerosis (severe)	6 (26)	2 (10)	0.22

ABMR: antibody-mediated rejection, IFTA: interstitial fibrosis/tubular atrophy.

## Data Availability

The data that support the findings of this study are available from the corresponding author upon reasonable request.
